# Determination of objective taste perception among Iranian medical sciences students during COVID‑19 pandemic in Yazd, Eastern Iran: a case-control pilot study

**DOI:** 10.1186/s12879-024-09897-7

**Published:** 2024-09-18

**Authors:** Samira Hajimaghsoodi, Elham Paydar, Fatemeh Owlia

**Affiliations:** 1 Department of Oral and Maxillofacial Medicine, School of Dentistry, Shahid Sadoughi University of Medical Sciences, Yazd, Iran; 2 Dentist, School of Dentistry, Shahid Sadoughi University of Medical Sciences, Yazd, Iran

**Keywords:** Saliva; taste threshold, COVID-19, Taste disorders, Dietary supplements

## Abstract

**Background:**

Taste disorders in patients suffering from COVID-19 were popular. Some people even after recovery report residual effects of loss of smell and taste. This study aimed to investigate the taste Perception of Iranian Medical Sciences students during the COVID-19 pandemic.

**Methods:**

The case-control study evaluated 50 Medical Sciences students with a history of COVID-19 infection, as confirmed by the Novel Coronavirus (2019-nCOV) CFX96™ Real-Time PCR Detection System. The other participants consisted of 50 volunteer students with COVID-19 negative. The taste perception was measured with 4 different concentrations of the basic tastes. The stimuli were applied to the protruded tongue. Subjects were asked to identify the researcher when they felt the taste. Data are expressed as frequency distribution and analyzed with the Chi-Square test (*P* < 0.05).

**Results:**

In this survey, 54% of participants were male and 46% were female, the mean age of participants was 22.96 ± 5 years. The results showed a significant difference in the sweet and bitter taste perception score according to the history of dietary supplement use. The bitter taste perception score declared a considerable difference since COVID-19 infection. There was no significant difference between the taste perception in the two studied groups for all 4 basic tastes according to gender, COVID-19 infection status, history of taste and smell disorders, and the elapsed time since COVID-19 infection.

**Conclusion:**

Our findings could provide important insights into taste perception. The history of dietary supplementation may influence how sweetness and saltiness are perceived. There was a noticeable difference in bitter taste perception depending on the time that had elapsed since the most recent COVID-19 infection.

## Introduction

SARS-CoV-2 is a coronavirus that causes the disease known as COVID-19. In addition to general symptoms, there is a surprisingly high prevalence of olfactory dysfunction (OD) and gustatory dysfunction (GD) in individuals with COVID-19 [1]. The COVID-19 pandemic was associated with the establishment of multiple guidelines for the dental profession [2]. Gustatory dysfunction is one of the typical symptoms in the course of COVID-19 disease [3]. Many symptoms in the oral cavity are attributed to COVID-19 disease; among them, oral pain and dysgeusia (taste disorders) are the most prevalent. Aphthous stomatitis and non-specific ulcerations are also mentioned [4, 5]. There are growing reports of oral lesions following COVID-19 vaccination [6]. COVID-19 vaccines have the potential to impact various biological systems while also stimulating the immune system. Some of these impacts are known as adverse effects. A review found that certain vaccines can have side effects that affect the mouth, including pemphigus vulgaris, bullous pemphigoid, herpes zoster, lichen planus, Stevens-Johnson syndrome, and Behçet’s disease [6, 7].

Taste has often been ignored as a sensory modality and is not considered as crucial as other senses. Patients may usually decline to disclose spontaneously whether they have taste disorders [8]. Although COVID-19 may be directly related to the dysfunction of taste receptor cells in the oral cavity, gustatory dysfunction in COVID-19 has largely been associated with olfactory dysfunction. Angiotensin-converting enzyme 2 (ACE2), the required entry receptor for SARS-CoV-2, is expressed in salivary glands and on oral epithelial cells. A probable mechanism for gustatory dysfunction by SARSCoV-2 is the high expression of ACE2 in the dorsum linguae. This phenomenon is induced through direct infection of taste receptor cells and thus influences stimulus transmission [9].

Saliva is a biomaterial that is readily available, and easy to collect. There is no need for special equipment for its collection. The collection process is inexpensive and non-invasive and can serve as a source of information for different diagnostic methods [10]. The taste system in humans has the potential to evaluate the food for nutrients and help us prepare the digestive tract for processing the introduced nutrients [11]. Humans usually recognize four basic tastes: sweet, salty, sour, and bitter. some literature suggests the existence of a taste modality that is responsive to fat through its breakdown product, fatty acids [12], and water through aquaporin (AQPs) [13, 14]. According to a recent review of the literature, the incidence rate of olfactory and taste dysfunction in COVID-19 patients has varied from 29.64 to 75.23% and 20.46–68.95%, respectively [15]. Many factors contribute to sense taste, such as the olfactory system, working habits, and physiological and psychosocial status [16].

It is an accepted fact that the COVID-19 virus affects taste perception. Many kinds of literature report different percentages of taste changes in different severities of COVID-19 disease [17–20]. Various studies have been done on taste perception at different times and in other study groups [1, 11, 17, 21, 22]. The innovation of this study was to conduct it on medical science students, who may be more aware of taste perception than the general population due to the nature of their field of study. The presence of a control group allowed for the differentiation between taste disorders associated with COVID-19 and those caused by other factors. This work provides a more accurate picture of taste changes caused by COVID-19. This pilot study was carried out at the closest point to the coronavirus pandemic in Iran and could serve as the basis for future studies over longer periods of COVID-19 infection. This study aimed to investigate the taste perception of Iranian Medical Sciences students during the COVID-19 pandemic.

## Subjects and methods

### Study design

In this case-control study, 117 medical science students with an age range of 18 to 26 years volunteered to participate. Clinical examinations accompanied by PCR tests diagnosed all of the COVID-19-positive patients. Real-Time Polymerase Chain Reaction (RT-PCR) was performed using the Novel Coronavirus (2019-nCOV) Nucleic Acid Diagnostic Kit (PCR-Fluorescence Probing) from Sansure Biotech (Changsha, China) on the CFX96™ Real-Time PCR Detection System (Bio-Rad Laboratories, Inc.), coupled with a thermal cycler according to the manufacturer’s instructions. Based on the history of positive SARS-CoV-2 PCR tests, individuals were divided into two groups: those with and those without a history of COVID-19 infection.

The inclusion and exclusion criteria.

Being an Iranian Medical Sciences student with, an age range of 18 to 26 years. Not having proved olfactory and gustatory disorders, a history of sinus operation, or radiotherapy or chemotherapy participation. If they are involved with a COVID-19 infection, the relevant specialist confirmed their infection by clinical examinations and PCR test.

Individuals were excluded if they smoked tobacco, took medications in the last month, or had allergies to the substances used in the study. Also, people who were unable to distinguish tastes or different concentrations from them were excluded from the study. Head trauma, smoking, cognitive impairments, and Parkinson’s disease were ruled out as causes of loss of smell or taste [23].

**Fig. 1:**
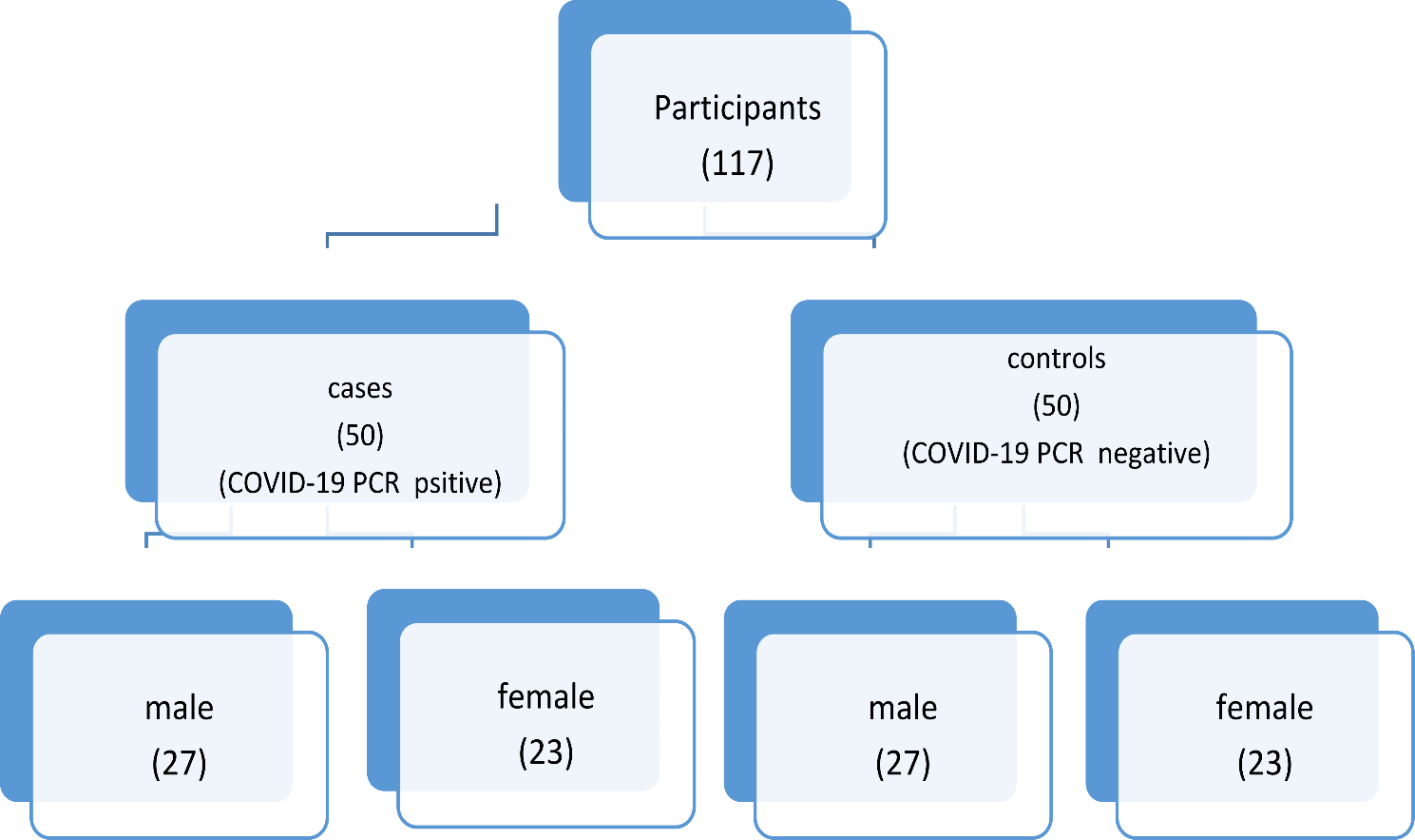
Flowchart of the study participants

### Study population

Medical Sciences students who tested positive for SARS-CoV-2 between June 3, 2022, and July 12, 2022, at the Yazd dental faculty were invited to participate in the study. The participants were divided into 2 groups: COVID-19-positive and COVID-19-negative groups. They were assessed in two groups, based on inclusion criteria. Demographic data of the participants were recorded. Students with a history of confirmed COVID-19 were asked questions about the time elapsed since the last infection, the history of taste or smell disorders at the time of infection, the severity of the infection, and the history of dietary supplement consumption (zinc, vitamin C, and vitamin D) during the period of infection or after. Data were recorded in the checklist.

The study was approved by the Ethics Committee of Yazd Shahid Sadoughi University of Medical Sciences, Yazd, Iran. (IR.SSU. DENTISTRY.REC.1401.010), and conducted by the Declaration of Helsinki. From all the subjects written informed consent was taken before participation.

### Data collection

Data was collected by a trained researcher in a way that is explained in detail below. The test of taste threshold was done at 9 to 10 in the morning and people should avoid eating and drinking, or using any kind of mouthwash for 2 h before that. The testing environment was controlled to minimize any distractions or external factors that could affect taste perception.

The taste test, performed at the Oral Medicine Department, is based on sterilized swabs soaked with the four substances (sodium chloride, citric acid, sucrose, and quinine hydrochloride) (Merck, Germany) to evoke the four basic taste qualities (salty, sour, sweet, and bitter). Each of the four tastes was presented at four different concentrations. These concentrations for each taste modality were used to examine the actual taste ability of patients. This valid concentration was similar to “taste strips” (Burghart, Wedel, Germany) [24]. The solutions (%) of the solvents are composed of 0.05, 0.1, 0.2, and 0.4 g/ml of sucrose for sweet concentrations 1–4 respectively. The concentrations for salty taste were 0.16, 0.04, 0.1, and 0.25 g/ml of NaCl. For sour taste 0.05, 0.09, 0.165, and 0.3 g/ml of citric acid were applied. The different concentrations for bitter taste were 0.0004, 0.0009, 0.0024, and 0.006 g/ml of quinine hydrochloride, respectively.

Sterile distilled water was used as the solvent, and taste solutions were freshly prepared on the day of the testing session. The solutions were stored at 25 ^o^C. The salt and sour stimuli were applied to the left and right sides of the tongue, respectively. To check the sweet taste threshold, the sweet swabs were placed on the anterior of the tongue, and for the bitter taste on the posterior of the tongue [17]. Participants properly were trained so that they understood the task and rating scales. Before applying the soaked swab, participants were asked to rinse their mouths with sterile distilled water. The taste presentations were randomized, and the sequence of stimulation was alternated. Subjects were asked to inform the researcher when they felt the taste.

### Sample size calculation and sampling method

The sample size formula was calculated considering the significance level of 5% and the test power of 80%. Based on the value of the standard deviation of the taste score (S = 0.85) obtained from previous studies, a minimum difference of 0.7 units in the average sweet taste score of 100 people is required to achieve statistical significance. Considering the possibility of dropping samples, 117 people were selected to participate in the study.

$$n = \frac{{{{\left( {{Z_{{\raise0.7ex\hbox{$\alpha $} \!\mathord{\left/{\vphantom {\alpha c}}\right.\kern-\nulldelimiterspace}\!\lower0.7ex\hbox{$c$}}}} + {Z_\beta }} \right)}^2}2{S^2}}}{{{{\left( {{X_1} - {X_2}} \right)}^2}}}$$

To address potential biases, the study considered the following factors: To prevent measurement bias, only one trained researcher performed the procedure to ensure consistent results. To avoid selection bias, the study was carefully designed and included clear criteria for selecting patients, which were detailed extensively.

### Statistical analysis

Statistical analysis was conducted using IBM SPSS Statistics ver. 25 (IBM Co., Armonk, NY, USA). The data used in this study were answers repeatedly collected from the same subject to the various types of stimuli. Results are expressed as frequency distribution. Differences were considered significant at *P* < 0.05. Chi-square and t-tests were used for data analysis.

## Results

The flow diagram of the study population is shown in Fig. 1. Data from 100 participants were included in the analyses. Data from seventeen participants (5 individuals from the case group and 12 individuals from the control group) were excluded due to protocol violations that occurred during test administration (8 persons), obscure medical history (5 persons), being a smoker (1 person) and having symptoms suspected of COVID-19 (3 persons). Finally, 100 participants (mean age 22.96 ± 5 years) completed the study. In each group, 27 persons were male, while 23 were female. The mean ± SD of age in the COVID-19-positive group was 22.7 ± 4.6 years with a range of 22 to 26 years. This value was 23.2 ± 5.5 years in the COVID-19-negative group with a range of 21 to 25 years. According to the statistical analysis, the two groups were similar in age (*P* > 0.05) and gender. None of the participants had a history of taking medication in the last month. All the participants who were in the COVID-19-positive group had mild clinical symptoms with no history of hospitalization or comorbidities. The taste perception of the basic tastes was reported in 4 grades (Table 1). There was no significant difference in the taste perception of main tastes based on the history of COVID-19 (*P* > 0.05).

**Table 1:** Groupwise comparison of taste perception at different concentrations in terms of PCR

Tastes	COVID-19 PCR results	concentrations 1 *N* (%)	concentrations 2 *N* (%)	concentrations 3 *N* (%)	concentrations 4 *N* (%)	Total *N* (%)	*P* value
Sweet	Positive	36(72)	6(12)	5(10)	3(6)	50(100)	0.606
	Negative	40(80)	4(8)	2(4)	4(8)	50(100)	
Salty	Positive	35(70)	10(20)	3(6)	2(4)	50(100)	0.319
	Negative	39(78)	6(12)	5(10)	0(0)	50(100)	
Sour	Positive	39(78)	8(16)	2(4)	1(2)	50(100)	0.396
	Negative	42(84)	8(16)	0(0)	0(0)	50(100)	
Bitter	Positive	33(66)	13(26)	4(8)	0(0)	50(100)	0.539
	Negative	34(68)	8(16)	5(10)	3(6)	50(100)	

Chi-square test

In Table 2, regardless of the history of COVID-19, a general comparison was made between men and women in the taste perception of different concentrations of the 4 main tastes. Chi-square analysis showed that there was no significant difference in the perception of any of the tastes between the two genders (*P* > 0.05).

The frequency distribution of taste perception scores based on the history of taste and olfactory disorders and dietary supplementation is presented in Table 3. It was shown that individuals who had dietary supplementation possess a significantly enhanced perception of the taste of both sweet (*p* = 0.048) and bitter taste (*p* = 0.039). There were no statistically significant differences in the perception of any tastes between individuals with a positive or negative history of taste and smell disorders at the time of COVID-19 infection (Table 3).

**Table 2:** Groupwise comparison of taste perception at different concentrations based on gender

Taste	Gender	concentrations 1 *N* (%)	concentrations 2 *N* (%)	concentrations 3 *N* (%)	concentrations 4 *N* (%)	*P* value
Sweet	Male	39(72.22)	5(9.25)	4(7.40)	6(11.11)	0.37
	Female	37(80.43)	5(10.86)	3(6.52)	1(2.17)	
Salty	Male	7(12.96)	5(9.25)	40(74.07)	2(3.70)	0.455
	Female	9(19.56)	3(6.52)	34(73.91)	0(0)	
Sour	Male	41(75.92)	11(20.37)	1(1.85)	1(1.85)	0.451
	Female	40(86.95)	5(10.86)	1(2.17)	0(0)	
Bitter	Male	35(64.81)	12(22.22)	5(9.25)	2(3.70)	0.935
	Female	32(69.56)	9(19.56)	3(6.52)	2(4.34)	

Chi-square test

**Table 3:** Groupwise comparison of taste perception in terms of the history of taste and olfactory disorders and the history of dietary supplementation

variables	status	Tastes	Score 1 *N* (%)	Score 2 *N* (%)	Score 3 *N* (%)	Score 4	total	*P* Value
Dietary supplementation	+	sweet	49(84.48)	2(3.44)	3(5.17)	4(6.89)	58	**0.048**
			27(64.28)	8(19.04)	4(9.52)	3(7.14)	42	
	+	salty	46(79.31)	7(12.06)	4(6.89)	1(1.72)	58	0.547
			28(66.66)	9(21.42)	4(9.52)	1(2.38)	42	
	+	sour	50(86.20)	6(10.34)	1(1.72)	1(1.72)	58	0.262
			31(73.80)	10(23.80)	1(2.38)	0(0)	42	
	+	bitter	41(70.68)	14(24.13)	3(5.17)	0(0)	58	**0.039**
			26(61.90)	7(16.66)	5(11.90)	4(9.52)	42	
Taste and olfactory disorders	+	sweet	9(56.25)	3(18.75)	3(18.75)	1(6.25)	16	0.320
			27(79.41)	3(8.82)	2(5.88)	2(5.88)	34	
	+	salty	10(62.5)	4(25)	2(12.5)	0(0)	16	0.379
			25(73.52)	6(17.64)	1(2.94)	2(5.88)	34	
	+	sour	12(75)	2(12.5)	2(12.5)	0(0)	16	0.177
			27(79.41)	6(17.64)	0(0)	1(2.94)	34	
	+	bitter	9(56.25)	4(25)	2(12.5)	1(6.25)	16	0.248
			24(70.58)	9(26.47)	1(2.94)	0(0)	34	

Chi-square test

Table 4 displays the taste perception in terms of the time elapsed since the last COVID-19 infection. The results showed that only the perception of the bitter taste had a slightly significant difference in terms of the time elapsed since the last COVID-19 infection (*P* = 0.048).

**Table 4:** Groupwise comparison of taste perception at different concentrations in terms of the time elapsed since the last infection with COVID-19

taste	Duration	concentration 1 *N* (%)	concentration 2 *N* (%)	concentration 3 *N* (%)	concentration 4 *N* (%)	*P* Value
sweet	Less than 3 months	3(75)	0(0)	1(25)	0(0)	0.147
	3 to 6 months	16(69.56)	2(8.69)	4(17.39)	1(4.34)	
	6 to 12 months	7(63.63)	4(36.36)	0(0)	0(0)	
	More than 12 months	10(83.33)	0(0)	0(0)	2(16.16)	
Salty	Less than 3 months	2(50)	2(50)	0(0)	0(0)	0.824
	3 to 6 months	14(60.86)	5(21.73)	2(8.69)	2(8.69)	
	6 to 12 months	10(90.90)	1(9.09)	0(0)	0(0)	
	More than 12 months	9(75)	2(16.66)	1(8.33)	0(0)	
Sour	Less than 3 months	2(50)	2(50)	0(0)	0(0)	0.177
	3 to 6 months	18(78.26)	3(13.04)	1(4.34)	1(4.34)	
	6 to 12 months	9(81.81)	2(18.18)	0(0)	0(0)	
	More than 12 months	10(83.33)	1(8.33)	1(8.33)	0(0)	
bitter	Less than 3 months	2(50)	2(50)	0(0)	0(0)	**0.048**
	3 to 6 months	14(60.86)	6(26.08)	2(8.69)	1(4.34)	
	6 to 12 months	7(63.63)	3(27.27)	1(9.09)	0(0)	
	More than 12 months	10(83.33)	2(16.66)	0(0)	0(0)	

Chi-square test

## Discussion

The quantitative and subjective assessment of the four taste qualities showed there were no significant differences in taste perception between the two groups studied. This was true for all four basic tastes, regardless of gender, COVID-19 infection status, history of taste and smell disorders, and the time elapsed since COVID-19 infection. The results indicated a significant difference in the sweet and bitter taste perception scores based on dietary supplementation. Additionally, there was a noticeable variation in the bitter taste perception score based on the duration since the participants’ COVID-19 infection.

The full scope and underlying pathophysiological mechanisms of gustatory dysfunction remain incompletely understood [1]. The etiologies of gustatory pathology include upper airway infections, viral cranial nerve disorders papillae or taste bud damage, and poor oral hygiene [25, 26].

Diagnosing a taste disorder can be challenging due to the numerous potential causes of taste loss. However, by further questioning patients about their symptoms, such as changes in taste, additional implications can be revealed. These implications include loss of appetite, weight loss, malnutrition, and ultimately, a lower quality of life [8]. Accurately measuring individual taste sensitivity, irrespective of COVID-19, is a complex task due to the many factors influencing taste perception.

Quantitative and subjective evaluations of the four basic taste qualities revealed that the most common score is 1, indicating sensitivity to the lowest tested concentration, with subsequent scores of 2, 3, and 4. Analysis shows that the distribution of taste perception frequencies does not significantly vary based on gender, past COVID-19 infection, or history of taste or olfactory impairments. Gustation is not an independent sensation. The olfactory system, which includes the nose, bulb, and olfactory cortex, plays a significant role in how a person perceives taste. Research has shown that 95% of taste disorders are caused by a loss of smell rather than a loss of taste [8]. As a result, assessing for a history of olfactory dysfunction was crucial.

While Förster et al. pinpointed infection severity, the overall severity of comorbidities, and female gender as crucial risk factors for post-COVID-19 conditions [18], the results of this study did not align with their findings [18]. In this study, it is crucial to include healthy controls who do not have COVID-19 so that they can be compared to participants of the same age and gender. The findings of this pilot study showed no statistically significant differences were observed in taste perceptions based on COVID-19 infection status or history of taste and olfactory disorders. Singer‑Cornelius indicated that most patients suffered from objective dysgeusia, especially in sour and salty taste [1].

Of course, significant differences were found in the frequency distribution of sweet and bitter taste perception in dietary supplement consumption history. Zinc and vitamins C and D play a role in improving taste perception in different ways. Zinc plays a role in taste perception by affecting neuronal processes. Specifically, it regulates the binding of amino acids to neurotransmitter receptors [27]. Vitamin C directly activates gustatory receptor neurons (GRNs) for sensing sweetness [28]. Vitamin D is known for its strong bitter taste and its ability to activate the G-protein-coupled TAS2R7, TAS2R10, and TAS2R14 taste bud receptors [29].

A possible factor for this divergence could be the severity of COVID-19 infection. In this survey, subjects only reported experiencing mild symptoms of COVID-19, with no cases requiring hospitalization [1, 18].

The obtained results revealed no significant difference in the taste perceptions for the four basic tastes based on the history of COVID-19 infection. This could suggest that the interaction between smell and taste, which improves relatively throughout the infection, might account for the transient nature of taste dysfunction [30]. Previous literature reports that 35.3–62% of COVID-19 patients experienced olfactory and taste disorders during their illness [21, 31, 32], with ageusia to hypogeusia ranging from 5.6 to 62.7% [33]. In two studies, 27.2% and 69% of COVID-19 patients exhibited taste disorders compared to healthy individuals, respectively, with a significant difference in the distribution of these disorders [34–36]. Parma et al. reported that nearly half of the COVID-19 patients experienced disorders in two or more taste qualities [33], indicating that more severe COVID-19 cases had a higher likelihood of olfactory and taste disorders [37]. The differences among the mentioned studies might be because of the use of various methods to assess taste disorders or ethnic differences.

Singer-Cornelius declared that the subjective perception of smell and taste dysfunction in COVID-19 patients may be overestimated, highlighting the superior accuracy and sensitivity of objective evaluations. A possible reason may be the awareness of patients about their positive SARS-CoV-2 test and the probable occurrence of smell and taste dysfunction in COVID-19 [1].

Accurate clinical evaluations and functional tests for olfactory and taste disorders are paramount. Many viruses affecting the upper gastrointestinal tract can induce olfactory and taste dysfunction, primarily through inflammation of the nasal mucosa. Angiotensin-converting enzyme 2 (ACE2) has been identified as a critical receptor for SARS-CoV-2 entry into host cells [38]. A study showed that cell populations expressing elevated levels of ACE2, such as those in the lungs, are at heightened risk of viral attack [39]. Xu et al. demonstrated through RNA transcript analysis that the ACE2 receptor is expressed in oral mucosa, with the tongue showing higher levels of expression than the cheek or gingiva [9]. This differential expression may explain the taste dysfunction observed in COVID-19 infections. In this study, standardized taste stimuli using laboratory-prepared solutions of consistent quality, a methodological advance over previous studies reliant on self-reports were used [40, 41]. Studies based solely on self-reports are influenced by individual feelings and perceptions, diminishing their value [42]. The results obtained using objective taste methods are higher than the subjective results. This is in agreement with previous studies [1, 43].

Independent of COVID-19, research has shown that women perceive sweet and bitter tastes more acutely than men, who exhibit a higher threshold for salty tastes. No significant gender difference was found in the perception of sour tastes [44]. Another study has indicated that women are more sensitive to olfactory and taste disorders [45], potentially due to hormonal and anatomical differences, including a higher number of fungiform papillae and taste buds in women [46, 47]. Despite these observations, the study identified no significant gender differences in taste thresholds for any of the four tastes, aligning with other studies’ findings [36, 48, 49]. This lack of difference could be due to the increased awareness of taste perceptions among the Medical Sciences Students compared to the general public.

A notable strength of this study was the uniform age of participants. Taste bud loss significantly affects individuals older than 40–45 years and reduces taste sensitivity with aging [46, 50]. Age is one of the primary indicators of hypogeusia in the general population [51]. Being younger participants in this study led to a comparatively lower baseline prevalence of taste loss than that found in past studies [32, 33].

When assessing taste perception regarding the time since the last COVID-19 infection, a little significant difference emerged for the bitter taste perception alone, revealing a reduced perception as more time had elapsed. This result diverges from the current literature, underscoring the need for further research [17, 49]. Bethineedi highlights that allelic variations of the T2R38 receptor could potentially impact patients’ innate immune response to SARS-COV-2 [52]. Niklassen believed that a loss of interaction between smell and taste on a CNS could be the reason for the transient character of taste disorder, which is relatively resolved during COVID-19 infection [49].

Asadi et al. reported significant increases in the taste thresholds for sweet, sour, and bitter tastes among COVID-19 patients, with the changes in bitter taste being more pronounced; conversely, the threshold for salty taste significantly decreased [17]. Hypersensitivity to salty taste was also noted by Parma et al., too [33]. However, it also has to be kept in mind, that due to the important role of sodium ions in the body’s hormones and electrolytes, the decrease in the saltiness threshold should be considered a serious warning [17]. Vaccination with Pfizer was found to impair the sweet taste sensation [53]. Cranial nerve dysfunction or taste bud damage likely contributes to increased thresholds through changes in saliva composition [17]. Another study highlighted a significant difference in the thresholds for sour and bitter tastes based on COVID-19 history [49].

A study involving approximately 3700 patients revealed that around 5.6% continued to experience a loss of smell, and 4.4% saw no improvement in taste after COVID-19 infection. Remarkably, within a month after infection, 79% of individuals reported an enhanced taste sensation, which increased to 98% after six months [54, 55]. A significant variation in the frequency distribution of sweet and bitter taste perceptions in the history of supplement consumption was observed in the study. This result supports the conclusions drawn by Lordan et al. regarding the positive effects of n-3 PUFA and probiotic supplements on managing and preventing COVID-19 symptoms. Specifically, it was found that zinc and vitamin C supplementation had a beneficial impact on the taste thresholds of hospitalized individuals [56].

To prevent misclassification bias, the interviewers were unaware of the participant’s case or control status. By employing a rigorous methodology, researchers can establish a reliable baseline for measuring taste sensitivity.

Considering the small sample size of this pilot study, its results should be interpreted with caution for future studies. Taste localization should be recognized as a multisensory phenomenon, transitioning from the outdated “tongue mapping” theory to a spatial approach to understanding taste perception [57]. The methodology employed in the study involved standardizing the application sites for taste solutions on the tongue for all participants, contrasting with previous research where solution placement varied [58]. Given the absence of significant differences in taste perception between participants, regardless of their COVID-19 PCR test results, it can be concluded that COVID-19 PCR test outcomes do not influence taste perception. This finding suggests that future research into taste perception may not require COVID-19 testing.

The reasons for the decline in participation were similar in both groups, mainly due to time constraints. This makes it unlikely that selection bias was present. However, there is a possibility of residual confounding caused by unmeasured variables, such as olfactory disorders. Of course, the measurement of taste disorder in the COVID-19 pandemic has an initial bias.

### Limitations of the study

A major limitation of this study is the absence of specialized examinations for olfactory functions, such as psychophysical tests or electrophysiological methods. Another limitation of the present study was the lack of evaluation of the dosage of dietary supplements. Considering the type of our study, which is not a cohort, and is only a pilot study, despite knowing the existence of initial bias, it was inevitable to fix it. Implementing more specific tests in studies with a larger sample size, could enhance sample screening and increase test accuracy and sensitivity.

## Conclusion

Our findings could provide important insights into how taste perception could be affected by COVID-19 infection and related factors. The history of dietary supplementation could play an important role in sweet and salt perception.

A notable difference in the bitter taste perception score was associated with the time elapsed since the last COVID-19 infection. These findings may assist healthcare professionals in identifying at-risk patients. Given the range of post-COVID-19 side effects, further research is warranted, focusing on the most disabling sequelae and the influencing factors.

## Data Availability

The datasets analyzed during the current study are available from the corresponding author upon reasonable request.
